# Supplementary material for: Learning to predict pain: differences in people with persistent neck pain and pain-free controls
Source: PeerJ. 2020 Jun 23;8:e9345. doi: 10.7717/peerj.9345 (PMC7319024; doi:10.7717/peerj.9345)
Supplement: Supplemental Information 1 [file peerj-08-9345-s001.docx]

**Appendix A**

Alex went to visit her physiotherapist after experiencing sustained neck pain. During her visit, Alex had to indicate whether she experienced pain at various positions of neck rotation. The physiotherapist noted down which positions were usually painful for Alex, and stored the notes in a folder. Unfortunately, a clumsy coffee spill caused the notes to get soaked in coffee. As a result, part of the physiotherapists notes became unreadable. <press space to continue>

We will now ask you to undertake a short learning task. During this task, you will be presented with images of Alex in various neck positions. After each presentation, you will be asked to what extent you EXPECT Alex to experience pain in the displayed neck position. You will indicate your expectation that Alex will have pain in that position by selecting a value on a 0-10 scale with your computer mouse (0=expect not at all, 10= expect very much). After selecting a value, you need to confirm your answer by pressing the spacebar. Afterwards, written information based on the physiotherapist his notes (‘PAIN’, ‘NO PAIN’, or ‘NOTES UNREADABLE’) will appear briefly on the centre of the screen, allowing you to learn whether that position was actually painful for Alex. <press space to continue>
