# Supplementary material for: Learning to predict pain: differences in people with persistent neck pain and pain-free controls
Source: PeerJ. 2020 Jun 23;8:e9345. doi: 10.7717/peerj.9345 (PMC7319024; doi:10.7717/peerj.9345)
Supplement: Supplemental Information 3 [file peerj-08-9345-s003.docx]

**Supplementary file**

To probe the degree to which differences in acquisition learning may mediate generalisation gradients, we performed a mediation analysis using Hayes PROCESS analysis.

A model 4 approach was employed (Figure 1), whereby X is Group (patient vs. control), Y is generalisation gradient (linear slope), and M is differential acquisition of pain-expectancy judgments at the last acquisition block (CS+ minus CS-). Thus, the direct effect of X on Y, and the indirect effect of X (via M) on Y could be assessed.

**Figure 1.** The mediation analysis model 4 (Hayes, 2017).

The total effect of X on Y was significant, and explained 22% of total variance (*b* = 0.81, t(58) = 4.1, p < 0.001, *r*^2^ = .22). The direct (*b* = 0.40, *SE* = 0.20, 95% CI [0.28 - 0.77]) and indirect effect X (*b* = 0.42, *SE* = 0.16, 95% CI [0.15 - 0.79]) were significant (i.e. confidence intervals do not include zero). These results suggest that both direct effect of Group and mediated effects of Group via acquisition learning contribute to the generalisation effect. Beta weights were remarkable similar, suggesting both pathways had similar predictive value. These results therefore indicate that at least part of the between-group difference in generalisation is independent on acquired differential learning.

Hayes, A. F. (2017). *Introduction to mediation, moderation, and conditional process analysis: A regression-based approach*. Guilford publications.
